# Vitamin D Toxicity Managed with Peritoneal Dialysis

**DOI:** 10.1155/2021/9912068

**Published:** 2021-06-28

**Authors:** Krystel Feghali, Kostas Papamarkakis, Jackson Clark, Neha Malhotra, Lanu Stoddart, Ibitoro Osakwe

**Affiliations:** ^1^Department of Endocrinology, Diabetes and Metabolism, University of Massachusetts Medical School, Baystate Medical Center, Springfield, MA, USA; ^2^Division of Nephrology, University of Massachusetts Medical School, Baystate Medical Center, Springfield, MA, USA; ^3^Division of Internal Medicine, University of Massachusetts Medical School, Baystate Medical Center, Springfield, MA, USA; ^4^Division of Pathology, University of Massachusetts Medical School, Baystate Medical Center, Springfield, MA, USA

## Abstract

Vitamin D deficiency is a global health issue that afflicts more than one billion children and adults worldwide. Vitamin D supplementation has increased over the years, whether through medical prescriptions, over-the-counter, or online purchasing. This is driven by a more recognized association between vitamin D sufficiency status and lower risk of cancer. In addition, more recently, it is used as a potential prophylactic and treatment for COVID-19 infection. This can lead to toxicity from overingestion. While rare, it has been reported in the literature. In this case report, we present a 75-year-old man with severe hypercalcemia secondary to vitamin D toxicity managed with peritoneal dialysis. He presented with biochemical evidence of hypercalcemia, acute kidney injury, and pancreatitis. Workup for his hypercalcemia led to the diagnosis of vitamin D toxicity as shown by a level greater than 200 ng/dL (Ref: 20–50 ng/mL) was confirmed by liquid chromatography-mass spectroscopy. Cornerstone medical management of hypercalcemia was provided which included aggressive intravenous fluid hydration, intravenous diuretics, calcitonin, bisphosphonate, and corticosteroid therapy. At every interruption of therapy, calcium levels trended upward. A thorough literature review yielded the finding of a sole case report from 1966 presented at the Third International Congress of Nephrology, in which peritoneal dialysis was used in the management of vitamin D toxicity and hypercalcemia. This modality is established to cause vitamin D deficiency. In collaboration with the nephrology team, 10 sessions of peritoneal dialysis were undertaken with resolution of hypercalcemia and downtrend in 25-hydroxyvitamin D levels as measured by dilution.

## 1. Introduction

Vitamin D deficiency and insufficiency are a global health issue that afflicts children and adults worldwide [1]. About 1 billion people have vitamin D deficiency, while 50% of the world's population has vitamin D insufficiency [2]. In the United States (US), the overall prevalence of vitamin D deficiency is estimated at 41.6% [3]. This global health issue has garnered medical attention for years, resulting in increased use of vitamin D supplementation. In 2013-2014, around 3.2% of the US adult population reported taking 4000 IU or more of vitamin D [4]. This may be due partly to the fact that vitamin D supplementation is readily available over-the-counter and through online purchasing. This upward trend in supplementation is also likely driven by a number of reasons, including the recognized association between vitamin D sufficiency status and lower risk of cancer [5–7]. More recently, vitamin D supplementation is used as potential prophylaxis and treatment for COVID-19 [8–10]. This raises the risk of complications including toxicity from overingestion. While rare, vitamin D toxicity has been reported in the literature. Herein, we present the case of a 75-year-old man with severe hypercalcemia secondary to vitamin D toxicity managed with peritoneal dialysis.

## 2. Case

**Figure 1:**
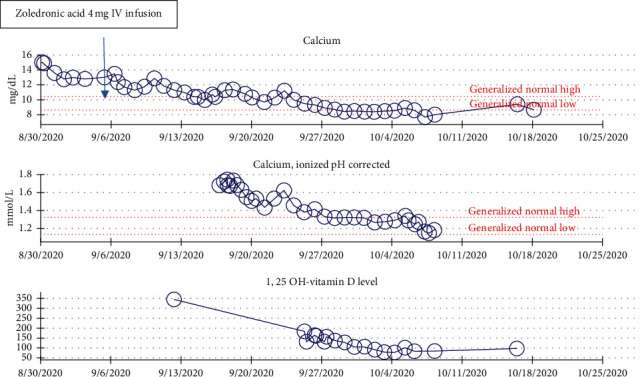
Trends of total serum calcium, ionized calcium, and 1,25-dihydroxyvitamin D levels during hospitalization.

**Figure 2:**
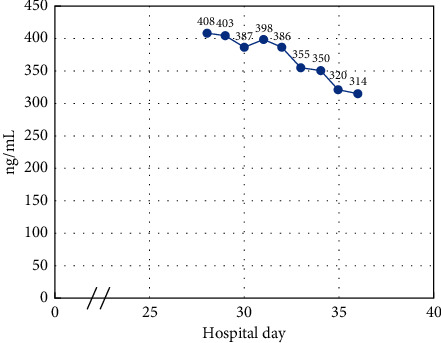
Serum 25-hydroxyvitamin D levels by dilution during hospitalization.

A 75 year-old male with a medical history significant for coronary artery disease, chronic kidney disease, hypertension, hyperlipidemia, heart failure with preserved ejection fraction, and a history of stroke presented to the emergency room with a two-week history of progressive altered mental status, generalized weakness, and confusion. On arrival, he was found to be profoundly hypertensive, with laboratory data showing an acute kidney injury, an elevated lipase above 2000, and a serum calcium of 15.0 (Ref: 8.6–10.5 mg/dL). Workup for his hypercalcemia revealed a normal intact parathyroid hormone (PTH) level of 20 pg/mL (Ref: 15–65 pg/mL), though repeat PTH following zoledronic acid infusion was low at 10 pg/mL (Ref: 15–65 pg/mL). While PTH-mediated hypercalcemia was initially entertained given the first nonsuppressed PTH, a sestamibi scan showed no evidence of parathyroid adenoma. The 25-hydroxyvitamin D level was elevated >200 ng/mL (Ref: 20–50 ng/mL), confirmed by liquid chromatography-mass spectroscopy, and the 1, 25-hydroxyvitamin D level obtained by immunochemiluminometric assay was elevated at 345 pg/mL (Ref: 19.9–79.3). 24-hour urine calcium was elevated at 0.34 gm/24 hours (Ref: 0.05–0.30 gm/24 hours). Other workup including an angiotensin-converting enzyme level, serum immunofixation, and parathyroid hormone related peptide levels were all within normal limits. A CT scan revealed no obvious evidence of sarcoidosis or lymphadenopathy. He was initially treated with intravenous fluids and diuretics, with initial transient improvement in his serum calcium levels. By hospital day 4, there was worsening of his hypercalcemia, and the endocrine service was consulted. More careful history revealed that the patient had bottles for both 5,000 IU and 50,000 IU of vitamin D at his home that were purchased over-the-counter, which he had been consuming daily for the past two years, consistent with the diagnosis of hypercalcemia secondary to vitamin D toxicity. He was given a dose of zoledronic acid 4 mg on hospital day 8 as well as a total of 7 doses of calcitonin, while maintaining normal saline infusions and furosemide, again with modest improvement in his serum calcium levels. Despite an initial response, the patient's calcium started to uptrend, and he was started on high-dose prednisone 60 mg daily and placed on a calcium restricted diet on hospital day 11. He received a total of around 63 liters of intravenous fluids throughout his 42 days hospitalization. Despite these measures, when trialed off fluids and diuretics, his calcium would uptrend. After a thorough review of the literature and an interdisciplinary consensus with the nephrology and transfusion medicine teams and discussion of the risk and benefits with the patient and his family, decision was made to start the patient on continuous cycling peritoneal dialysis (CCPD) to treat his vitamin D toxicity. After the patient provided informed consent, a peritoneal dialysis catheter was placed on hospital day 27, and he was started on peritoneal dialysis. He completed 10 sessions of CCPD. His progress was monitored with total serum calcium, ionized calcium, and both 25-hydroxyvitamin D by dilution and 1, 25-hydroxyvitamin D levels after each dialysis session (Figures 1 and 2). The nephrology team measured 25-hydroxyvitamin D and 1, 25-hydroxyvitamin D levels in the peritoneal dialysis effluent fluid.

We used 24 hours CCPD with 1.5% dextrose solutions with 1.25 liters dwells cycling every 4 hours for a total of 10 sessions over 10 days. Our patient had a serum 25-hydroxyvitamin D level of about 400 ng/mL. Considering that an estimated 30% of total body vitamin D is circulating in the serum (with the rest distributed in adipose tissue, muscles, and other tissues) [11] and assuming a total blood volume of 4.0 liters, the patient at any one time had about 1,600,000 ng of circulating 25-hydroxyvitamin D.

His peritoneal dialysis effluent 25-hydroxyvitamin D ranged 60–77.8 ng/mL of effluent. With a fixed effluent of 1.25 liters, we effectively cleared about 75,000–97,000 ng of 25-hydroxyvitamin D with each exchange, corresponding to about 3,000–3,880 IU.

Eight days following discharge, blood work revealed a calcium level maintained in the normal range at 9.4 mg/dL and a 25-hydroxyvitamin D level of 461 ng/mL. His kidney function had also returned to baseline.

## 3. Discussion

**Figure 3:**
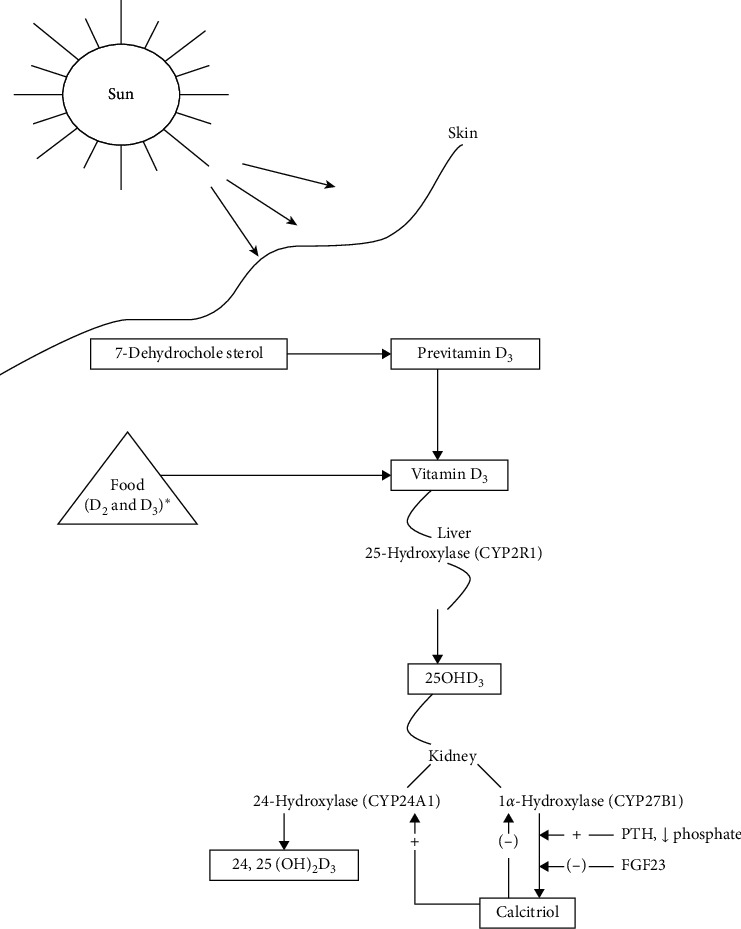
Pathway of vitamin D synthesis and metabolism (adapted from dietary reference intakes for calcium and vitamin D [14]).

Vitamin D was first described as a vitamin in the 20^th^ century and is now recognized as a prohormone. A unique feature of vitamin D is that it is synthesized by the human body through direct sunlight exposure. Interestingly, prolonged exposure to UV light does not lead to toxic amounts of vitamin D3 because previtamin D3 and vitamin D3 are photoconverted to inactive metabolites [12, 13]. As shown in Figure 3, 7-dehydrocholesterol is converted to previtamin D3 through UVB in the skin. Previtamin D3 is then converted to vitamin D3 (cholecalciferol) through the effects of heat. Vitamin D3 is converted by 25-hydroxylase (CYP2R1) in the liver to 25-hydroxyvitamin D, the major circulating form of vitamin D. It circulates and binds to the vitamin D binding protein (DBP). In the kidney, 1*α*-hydroxylase converts 25-hydroxyvitamin D to 1,25-dihydroxyvitamin D (calcitriol), the active form. The renal synthesis of 1, 25-dihydroxyvitamin D is counter regulated by 24-hydroxylase that converts 25-hydroxyvitamin D into inactive 24, 25-dihydroxyvitamin D. Vitamin D is a fat-soluble vitamin and stored in adipose tissue. Reuptake of vitamin D in the kidneys occurs through binding to vitamin D binding protein. Thus, the excretion of vitamin D metabolites is primarily through the bile into the feces [14].

Vitamin D excess is described with certain inactivating mutations of CYP24A1 encoding vitamin D-24-hydroxylase [15], and more commonly, although infrequently, in situations of exogenous supplementation.

The daily upper limits for vitamin D supplementation are outlined in Table 1 [14]. From the year 2000 through June 30th, 2014, the National Poison Data System received reports of 25,397 human exposures to vitamin D. Between the years 2000 and 2005, there was an average of 196 reported cases per year. By the end of 2014, there was a 1600% increase in exposure, with 4535 cases recorded [16].

Hypercalcemia is a biochemical manifestation of vitamin D toxicity (VDT). Signs and symptoms of VDT are due to the underlying hypercalcemia and include confusion, polyuria, polydipsia, anorexia, vomiting, and muscle weakness. Chronic intoxication may cause nephrocalcinosis, bone demineralization, and pain. The therapeutic approach to this non-PTH dependent hypercalcemia is universal. Consistently seen across several case reports and case series over the past 5 years [17–23], management of hypercalcemia secondary to hypervitaminosis D consisted of aggressive intravenous fluid resuscitation, diuretic therapy, calcitonin, and bisphosphonate therapy (alendronate, pamidronate, or zoledronic acid). Corticosteroid therapy (prednisone or prednisolone) has also been the cornerstone of management of 1,25-dihydroxyvitamin mediated hypercalcemia. It lowers the calcium level by reducing intestinal calcium absorption by decreasing transcellular active transport processes, increasing urinary excretion of calcium and altering the hepatic vitamin D metabolism to favor synthesis of inactive metabolites [24]. Second line therapy of VDT includes phenobarbital, ketoconazole, or aminoquinolones [24]. In addition, August et. al described the use of hydroxychloroquine 400 mg daily for hypercalcemia secondary to VDT in a 54-year-old man with kidney failure [25]. Rifampin and phenytoin have also been used in the management of VDT as they both induce CYP3A4 which metabolizes 25-hydroxyvitamin D into inactive 4*β*,25-dihydroxyvitamin D. This is the alternative pathway for vitamin D inactivation in patients with CYP24A1 mutations [26].

Despite initial response to medical management with intravenous hydration, diuretics, calcitonin, bisphosphonate, and corticosteroid therapy and notwithstanding adherence to a calcium restricted diet provided through the assistance of the dietary and nutritional services, our patient experienced rebound hypercalcemia at every interruption in management. After almost a month, our focus shifted to efforts to eliminate vitamin D from his serum. Peritoneal dialysis is established to cause vitamin D deficiency. Alwakeel et al. presented data from 27 patients who had been on peritoneal dialysis for more than six months. Blood was sampled for 25-hydroxyvitamin D levels. The majority of patients (59.2%) had levels below 15 nmol/L (6 ng/mL) [27]. Literature review yielded a sole case report from 1966 presented at the Third International Congress of Nephrology, in which peritoneal dialysis was used to clear vitamin D and calcium in the case of a 54-year-old woman with hypercalcemia secondary to hypervitaminosis D. This latter patient had received more than 100 million units of vitamin D orally and intramuscularly over a period of 3 1/2 years for vitamin D resistant rickets [28].

## 4. Conclusion

Peritoneal dialysis is often used as a means of addressing hypercalcemia; however, since 1966, it has not been used to our knowledge for the treatment of hypervitaminosis D. In the case we presented, we were able to safely use peritoneal dialysis to clear 25-hydroxyvitamin D from the plasma resulting in faster resolution of hypercalcemia.

The increase in our patient's 25-hydroxyvitamin D level after discharge can be explained by the release of vitamin D from adipose tissue stores. Fortunately, his calcium level has remained in the normal range, and he remains on a restricted calcium diet.

This case, in addition to providing an alternative option for the treatment of vitamin D toxicity, raises concern regarding vitamin D reserves in patients who routinely undergo peritoneal dialysis for renal replacement therapy as Alwakeel et al. described in 2014 [27]. According to our calculation, a single cycle of peritoneal dialysis leads to the extraction of approximately 75–90 k ng of vitamin D. This urges the question about the systemic effects of chronic vitamin D losses in this group of patients. More research is needed in this field to study these outcomes.

Sequalae of vitamin D toxicity remain a concern. Nephrocalcinosis defined as a generalized deposition of calcium salts in the kidney is a well-known but rare complication of VDT as seen in a 14-year follow-up report of an 8-month-old child [29]. Vascular calcification, resulting in increased cardiovascular risk, is another complication of hypercalcemia and vitamin D toxicity. Reversal of VDT and hypercalcemia has resulted in resolution of vascular calcifications [30].

## Figures and Tables

**Table 1:** Daily upper limit for vitamin D supplementation (adapted from dietary reference intakes for calcium and vitamin D [14]).

Life stage group	UL
Infant	
0–6 m	1,000 IU (25 *μ*g)
6–12 m	1,500 IU (38 *μ*g)

Children	
1–3 y	2,500 IU (63 *μ*g)
4–8 y	3,000 IU (75 *μ*g)

Male	
9–13 y	4,000 IU (100 *μ*g)
14–18 y	4,000 IU (100 *μ*g)
19–30 y	4,000 IU (100 *μ*g)
31–50 y	4,000 IU (100 *μ*g)
51–70 y	4,000 IU (100 *μ*g)
>70 y	4,000 IU (100 *μ*g)

Female	
9–13 y	4,000 IU (100 *μ*g)
14–18 y	4,000 IU (100 *μ*g)
19–30 y	4,000 IU (100 *μ*g)
31–50 y	4,000 IU (100 *μ*g)
51–70 y	4,000 IU (100 *μ*g)
>70 y	4,000 IU (100 *μ*g)

Pregnancy	
14–18 y	4,000 IU (100 *μ*g)
19–30 y	4,000 IU (100 *μ*g)
31–50 y	4,000 IU (100 *μ*g)

Lactation	
14–18 y	4,000 IU (100 *μ*g)
19–30 y	4,000 IU (100 *μ*g)
31–50 y	4,000 IU (100 *μ*g)

Note: IU, international unit.

## Data Availability

The data used to support the findings of this study are available from the corresponding author upon request.
